# Relationship between serum ferritin and growth status of pediatric transfusion dependent thalassemia 

**DOI:** 10.22088/cjim.14.3.425

**Published:** 2023

**Authors:** Andi Cahyadi, I Dewa Gede Ugrasena, Mia Ratwita Andarsini, Maria Christina Shanty Larasati, Raden Muhammad Zulfan Jauhari, Diah Kusuma Arumsari

**Affiliations:** 1 Department of Child Health, Faculty of Medicine, Universitas Airlangga/Dr. Soetomo General Academic Hospital, Surabaya, East Java, Indonesia

**Keywords:** Pediatric transfusion-dependent thalassemia, Serum ferritin, Growth status.

## Abstract

**Background:**

Growth retardation is a long-term complication in pediatric transfusion-dependent thalassemias (TDTs), presented as short-stature and upper body segment shortening. The cause of this condition was chronic hypoxia, iron overload, endocrinopathy, inadequate transfusion, and iron chelation. We analyze the relationship between ferritin level and growth status of pediatric TDTs.

**Methods:**

This was a cross-sectional study on pediatric TDTs aged 2-18 years old at Dr. Soetomo General Academic Hospital Surabaya, Indonesia conducted in 2020. They required blood transfusion every 2-4 weeks. We evaluated the ratio of upper/lower body segments, weight for age Z-score (WAZ), height for age Z-score (HAZ), and body mass index (BMI) Z-score, based on CDC growth chart as growth status parameters. Serum ferritin was checked every three months to determine iron overload and iron chelation (deferiprone, deferasirox and deferoxamine). We used Spearman correlation and Mann-Whitney U test to analyze between variables (α=0.05).

**Results:**

We enrolled 15/29 males with median age 10.5 years. Serum Ferritin had negative correlation with the ratio of upper/lower body segments (rho=-0.552; P=0.002), but not for HAZ (rho=-0.078; P=0.694), WAZ (rho=-0.186; P=0.342), BMI Z-score (rho=-0.089; P=0.653) especially if serum ferritin was above 2500 µ/L. In deferiprone group (n=8), the WAZ (P=0.034) and BMI Z-score (P=0.031) were lower; but the ratio of upper/lower body segments was greater (P=0.039) than the deferasirox group.

**Conclusion:**

Growth retardation was more visible in pediatric TDTs with high ferritin and in deferiprone group. The height and the ratio of upper/lower body segments of the body were more affected.

Growth retardation almost always occurs in pediatric transfusion-dependent thalassemias (TDTs), presented as short stature, decreasing growth rate and the upper and lower segments ratio of the body, and impaired bone age at 6 to 7 years of age. Growth retardation becomes more severe when the child fails to achieve a growth spurt at 9 to 10 years old. 

Most pediatric TDTs fail to reach the final height as their counterpart (1-3). Chronic anemia, iron overload, and iron chelation toxicity are the main factors causing growth retardation in pediatric TDTs. Other factors are lack of protein-calorie energy, micronutrient, and macronutrients deficiency (3). Endocrinopathy caused by iron overload leads to growth hormone deficiency, hypogonadism, and hypothyroidism (1, 3, 4). A Malaysian study reported that 64.6% of pediatric TDT patients had one endocrine disorder (5). 

Based on the 2007 National Health Survey, the prevalence of thalassemia in Indonesia is 1.5 per 1000. Three provinces with a high prevalence of thalassemia are Nanggroe Aceh Darussalam (13.4‰), DKI Jakarta (12.3‰), and South Sumatra (5.4‰). Thalassemia carriers occurred in 5.41% of the general population and 28% of families with thalassemia in 2008-2014. The height, weight, and body mass index (BMI) in pediatric TDTs vary, partly normal, below, and above average. Increasing age would decrease height for aged Z-score and BMI Z-score, but height for aged Z-score is more affected. Height velocity decreased more rapidly than weight loss (3, 4, 6). The prevalence of failure to reach height occurs in 33-70% of children (3, 6-9). Stunting has started at the age of 5 to 10 years but increases sharply with increasing age (2). The spine growth will slow down during infancy then progressively decline, especially in boys. Bone age and bone maturation in children with TDT will also experience a delay (3, 4). Ferritin level in TDTs children who have stunted was higher than children with average growth (3, 6, 9-11). Ferritin levels are closely associated with growth disorders in pediatric TDT, especially short stature and decreasing in percentile or height Z-score for age (9, 11). Early identification of iron overload, growth retardation, Iron chelation offerings can be used to improve the quality of life of children with thalassemia. This study aimed to discuss the relationship between serum ferritin and growth status of pediatric transfusion-dependent thalassemia. 

## Methods

This cross-sectional study studied pediatric TDT aged 2-18 years, who routinely undergo blood transfusions at Hematology Oncology Outpatient Clinic, Department of Pediatrics, Dr. Soetomo General Academic Hospital, Surabaya, East Java Indonesia in 2020. This research ethics was approved by the Health Research Ethics Committee in our institution with the ethical number 1980/KEPK/IV/2020. Thalassemia was characterized by the decreased or absent synthesis of one or more of the normal-globin chains with the most relevant type was α and β-thalassemias. Transfusion dependent thalassemias (TDTs) required a regular blood transfusion to survive. This category includes patients with β-thalassemia major, severe HbE/β-thalassemia, transfusion-dependent HbH disease or HbH hydrops, and surviving HbBart’s hydrops (12). The TDTs usually required blood transfusions at least 2-4 weeks to maintain hemoglobin levels above 9-10.5 g/dL. They usually get blood transfusions from less than two years old. The pediatric hematologist made the diagnosis of TDTs, based on hemoglobin electrophoresis. We used the proportion data formula to calculate the sample size with an error rate of α=0.05, power of test 80% for a two-sided test, and consecutive sampling.

The chronological age was grouped into less than ten years and above ten years. Anthropometric examination for body weight, height, length of the upper and lower segments of the body uses standard methods. Children take off footwear and accessories (watches, hats, jackets, weight bracelets, glasses, and jackets). The body weight was measured using the Seca® brand weight scale expressed in kilograms (kg). Height was measured by standing straight, eyes straight ahead, the arms at the sides, the knees are upright and should not be bent using the Seca® brand stature meter, expressed in centimeters (cm). The length of the upper body was obtained by subtracting the height from the lower body segment. The lower body segment was the distance from the symphysis pubis to the floor in a patient standing erect against a wall. The ratio was measured by dividing the upper segment value by the lower segment value of the body (13). Body mass index (BMI) calculation was the ratio of body weight (kg) and height squared (meters) expressed in kg/m^2^. The body weight, body height, and BMI would be converted into the standard of deviation (SD) Z-score values and compared with the growth curve from the Center for Disease Control (CDC) in 2000 using the CDC Growth calculator for 2 to 20 years (14). The anthropometric parameters showed for weight for age Z-score (WAZ), height for age Z-score (HAZ), and BMI Z-score (BMI Z-score). The normal value of the Z-score was between -2 and +2 standard deviation.

The routine check at each visit was a complete blood counts evaluation using the Sysmex® XP-100 Hematology Analyzer. The pediatric TDTs received blood transfusions with a post-transfusion hemoglobin target of 11-12 g/dL using leukoreduced PRC (12). The volume in each transfusion did not exceed 10 mL per kg body weight per day in the outpatient clinic setting. Serum ferritin levels were analyzed using the Enzyme-Linked Fluorescent Assay (ELFA) technique at the Clinical Pathology Laboratory, Dr. Soetomo General Academic Hospital. Samples were taken from 3 mm of peripheral blood in a vacuum tube without anticoagulant. The mean ferritin (µg/L) was the average of at least three measurements in the last year.

We gave iron chelation if the ferritin levels were more than 1000 µg/L or they had been receiving blood transfusions more than ten times or had obtained signs of iron overload according to the guidelines of the Thalassemia International Federation in 2014. There is three iron chelation. They were oral deferiprone 75-100 mg per kg body weight in three divided doses (syrup 500 mg/5 ml and tablets 500 mg), oral deferasirox 10-20 mg/kg body weight once a day, and deferoxamine 20-40 mg per kg body weight intravenously. The dosage and replacement of chelation iron depend on the latest ferritin results and the policy of a pediatric hematologist.We did not evaluate other factors that can cause growth retardation, such as endocrinopathy (growth hormone deficiency, hypogonadism, hypothyroidism), chelation toxicity, nutritional deficiency (vitamin D, zinc and carnitine deficiencies), chronic liver disease and psychosocial stress. Subjects who had been treated for endocrinopathy were excluded from the study sample. We also did not measure puberty stage. We used the software of the statistic program for social science (SPSS) 18 for windows. The relationship between ferritin, HAZ, WAZ, BMI Z-score, and the ratio of upper/lower body segments were analyzed using the Spearman rho correlation test. The differences in anthropometric parameters based on the serum ferritin group, gender, age group, and type of chelating iron were analyzed using the Mann-Whitney U test. The differences in proportions data were analyzed using the chi-square test. We used a significance level of p <0.05 for the two-tailed sides.

## Results

A total of 29 children meet the criteria in this study. They included 15 boys with a median age of 10.5 years old, and 15 of 29 children were above ten years. Most of them suffered from β-thalassemia (26/29) and three children with α-thalassemia. They had suffered thalassemia for a median of 8.8 years (table 1). There was negative correlation between age and HAZ (P=0.004 and rho= -0.520), WAZ (P=0.002 and rho=-0,500) and BMI Z-score (P=0.013; rho=-0.455). The WAZ, HAZ, and BMI Z-scores between boys and girls did not show any significant difference. In pediatric TDT above ten years old, there were significantly lower than in children aged less than ten years (p<0.05). The lengths of the upper and lower segments were not significantly different between boys and girls and age categories. The ratio of upper/lower body segments was greater in younger children below ten years than children above ten years old (P=0.023), presented in table 2. 

The main iron-chelating agents were deferiprone (n=20), deferasirox (n=8), and deferoxamine in one child. WAZ and BMI Z-scores in the deferiprone group were lower than in the deferasirox group with P=0.034 and P=0.031 but not for HAZ, presented in table 3. The upper and lower segments in the deferiprone group were shorter than in the deferasirox group with p<0.001. The ratio of upper/lower body segments in the deferiprone group was higher than in the deferasirox group with P=0.039. The median ferritin in the deferiprone group was lower than the deferasirox group. Serum ferritin in one child with deferoxamine was 10,702 µg/L. Twelve (37.9%) children had ferritin levels less than 2500 µg/L. Chronological age had a positive correlation with serum ferritin (rho=0.485; P=0.009) based on table 4. The WAZ, HAZ, and BMI Z-scores did not correlate with serum ferritin. The length of the upper body segment did not correlate with ferritin, but the lower body segment correlated with serum ferritin (rho=0.380; P=0.046). The ratio of upper/lower body segments had a negative correlation with the serum ferritin (rho=-0.552; P=0.002).

**Table 1:** Subject characteristics of TDT children

**Characteristics**	**N=29**
**Sex (n)** **Boys, n** **Girls, n**	15 14
**Age at diagnosis, median (range) year**	1,8 (0,08-6,08)
**Chronological age, median (range) year**	10,4 (2,58-17,67)
**Duration of suffering thalassemia, median (range) year**	8,8 (0,42-16,25)
**Age categories** **< 10 years, n** **>10 years, n**	14 15
**Thalassemia type** **α-Thalassemia, n** **β-Thalassemia, n**	3 26

**Table 2:** Anthropometrics parameters base on sex and age classification

**Parameters**	**Boys** **Median (range)**	**Girls** **Median (range)**	P*
**WAZ (weight for age Z-score)**	-1.7 (-3.10 to -0.33)	-2.1 (-3.10 to 3.10)	0.354
**HAZ (height for age Z-score)**	-2.1 (-4.05 to -0.12)	-2.0 (-8.02 to 3.10)	0.793
**BMI (body mass index) Z-score**	-2.1 (-4.05 to -0.12)	-2.5 (-8.02 to 3.10	0.295
**Upper segment, cm**	63.0 (53.0 to 85.0)	61.5 (48.0 to 85.0)	0.458
**Lower segment, cm**	59.0 (42.0 to 85.0)	62.0 (39.0 to 78.0)	0.678
**Upper/lower segment ratio**	1.0 (0.92 to 1.34)	1.1 (0.91 to 1.23)	1.000
	< 10 years old	>10 years old	P*
**WAZ (weight for age Z-score)**	-1.1 (-3.10 to 3.10)	-3.1 (-3.10 to 1.89)	0.021
**HAZ (height for age Z-score)**	-1.6 (-4.05 to -0.12)	-3.3 (-8.02 to 3.10)	0.036
**BMI (Body mass index) Z-score**	-1.6 (-4.05 to -0.12)	-3.3 (-8.02 to 3.10)	0.023
**Upper segment, cm**	58.8 (48.0 to 63.0)	70.0 (56.0 to 85.0)	<0.001
**Lower segment, cm**	55.5 (39.0 to 61.0)	70.0 (54.0 to 85.0)	<0.001
**Upper/lower segment ratio**	1.1 (0.92 to 1.34)	1.0 (0.91 to 1.20)	0.023

*Mann-Whitney U test

**Table 3:** Anthropometrics parameters base on iron chelation

**Characteristics**	**Deferiprone** **N=20**	**Deferasirox** **N=8**	**P**
**Boys** **Age categories**	9	6	0.221^a^
**< 10 years, n**	9	5	
**>10 years, n**	11	3	0.678^a^
**WAZ, median (range)**	-1.8 (-3.10 to 1.89)	-1.6 (-3.10 to 3/10)	0.034^b^
**HAZ, median (range)**	-2.1 (-8.02 to 3.10)	-2.3 (-4.05 to -0.56)	0.060^b^
**BMI Z-score, median (range) kg/m2**	-1.0 (-2.66 to 2.212)	-0.8 (-1.87 to 3.10)	0.031^b^
**Upper segment, median (range) cm**	62.0 (48.0 to 85.0)	71.5 (54.0 to 85.0)	<0.001^b^
**Lower segment, median (range) cm**	57.0 (39.0 to 78.0)	70.0 (48.0 to 85.0)	<0.001^b^
**Upper/lower segment ratio**	1.1 (0.97 to 1.34)	1.0 (0.92 to 1.18)	0.039^b^

^a^Fisher Exact test, ^b^Mann-Whitney test

**Table 4:** Correlation between anthropometric evaluation and serum ferritin in pediatric TDT

**Parameter**	Rho*	P*
**Age**	0.485	0.009
**WAZ (weight for age Z-score)**	-0.078	0.694
**HAZ (height for age Z-score)**	-0.186	0.342
**BMI Z-score, kg/m2**	-0.089	0.653
**Upper segment, cm**	0.123	0.532
**Lower segment, cm**	0.380	0.046
**Upper/lower segment ratio**	-0.552	0.002

*Spearman rho correlation

## Discussion

This study identified a growth status in pediatric TDTs, half of the cases were TDTs above ten years, and 90% were β-thalassemia cases. Increasing age would increase the risk of short-stature and decrease BMI Z-score, especially in children older than ten years old. Decelerating of height velocity was more pronounced than decelerating of body weight. Increasing age had a negative correlation with the ratio of the upper and the lower segments of the body. Abnormal growth status occurred after ten years old, in the deferiprone group with higher ferritin levels. Boys and girls had the same median WAZ, HAZ, and BMI Z-scores. The HAZ in children above ten years old was lower than in children under ten years old. As many as 47% of children with major beta-thalassemia aged 4-15 years are underweight in Egypt (7). 

Underweight accounted for 77% of TDT of children less than 18 years old in India (9). About 43% of children have a low BMI (7). If bodyweight and height decreased proportionately, BMI should be constant. However, in pediatric TDTs, the BMI Z-score also declined. It showed that the height decrease is more dominant than the decrease in weight. Moiz et al. (2018) reported that the median HAZ was low at -2.69 (1.46 to -3.80) while in this study, the median was -1.7 (-3.10 to -0.33) for boys and -2.1 (-3.10 to 3.10) in the girl. Increasing age would decrease HAZ and BMI Z-score, but HAZ is more affected, indicating that slowing of height rate decreased more than slowing of body weight (2, 4, 6). 

The HAZ in this study was still better than the research in Pakistan (2). The number of pediatric TDTs who had a HAZ less than minus 2 in a standard deviation was higher at above ten years of age (73%). The prevalence of failure to achieve normal height in children with thalassemia was quite high. It reached 33-70% of pediatric TDTs (2, 5-9, 11). Short stature was the major endocrine disorder in pediatric TDTs aged 2-25 years in Malaysia which reached 40%, and 81.8% occurred aged 10-19 years (5) and 65.4% occurred in aged 5-17 years in Pakistan (2). The decrease in HAZ values can be caused by changes in the length of the upper and lower segments of the body. Apart from short stature, children with TDTs in Malaysia also experienced spinal shortening. Apart from chronic endocrinopathy and hypoxia, pediatric TDT would also experience shortening the bone length. It becomes shorter than normal children (4). Differences in HAZ in diverse countries occurred via genetic differences, iron chelation toxicity, iron overload, and endocrinopathy. Quality of care, chronic hypoxia associated with recurrent anemia, delay in getting blood transfusions, and the availability of iron chelation and regularity of taking medication also affected the growth of children with thalassemia. Growth retardation in thalassemia, caused by a growth hormone deficiency, reached 26.6% of cases (15). 

Children less than ten years old had lower upper and lower segment lengths than children above ten years old. The differences occurred in pre-pubertal and post-pubertal, especially in children with suboptimal iron chelation and due to abnormal growth of vertebral bones (3). About 15-40% of pediatric thalassemia majors will experience a body disproportion between upper and lower segments. It suggested that short stature was not proportionally due to shortening of the upper body segments, but changes also occurred in the vertebral bodies. Spinal growth will slow down during infancy, then progressively decline, especially in boys. Bone age and bone maturase in pediatric TDTs will also experience delay (3, 4). Growth retardation occurred in 41.1% of 4418 patients with beta-thalassemia, mainly males (15). TDTs patients with short stature had lower standard deviations of sitting height, standing height, and subischial leg length. The mean standard deviations of sitting height and the mean subischial leg length in TDTs patients with short stature were due to disproportionate truncal shortening (10).

Adequate blood transfusion and optimal iron chelation have improved growth in pediatric TDTs. However, growth retardation could still occur in childhood and young adulthood (16). The management of thalassemia in Southeast Asia, including Indonesia, is currently not uniform, as is the preparation of iron chelators. The accessibility of iron chelation is also different, the impact on the growth and development of children is also different (17). Deferiprone was the most iron chelator in our institution. The WAZ in the deferiprone group was lower than the deferasirox but the opposite in the BMI Z-score. The length of the upper and lower segments in the deferiprone group was shorter compared to the deferasirox group. The ratio of upper/lower body segments was higher in the deferiprone group than in the deferasirox group. There was only one child taking deferoxamine. All growth parameters were retarded. Adequate iron chelation has promoted growth in pediatric TDTs, but TDTs treating with desferoxamine still has short stature. They also had a disproportion between the upper and lower body segments (1, 3). 

Administration of iron chelation before puberty could reduce the prevalence of endocrinopathy and achieve adult standing height. Children taking deferiprone had a lower median ferritin level than deferasirox. The median value of serum ferritin levels between boys and girls did not differ significantly, as did the type of thalassemia (6). Ferritin levels in children above ten years of age were higher than below ten years. Only 41.4% of children had serum ferritin levels less than 2500 µg/L. The results of this study are similar to Rathaur et al. (2020). He reported 45.7% lower than Fadlyana et al. (2017) who reported 77%. About 3% of children with TDTs had serum ferritin less than 2000 µg/L in Pakistan and did not differ between men and women (2). The proportion of pediatric TDTs who had serum ferritin levels less than 2500 µg/L increased from 35% to 45% after using deferasirox for 12 months (18). A meta-analysis of nine clinical trials showed that 75.5% of children with iron overload experienced a decrease in serum ferritin after treatment with deferiprone 75 mg/kg/day. The mean serum ferritin decreased 23.5% from the initial serum ferritin after 16 months of therapy (19). However, deferiprone and deferasirox were equally effective in lowering serum ferritin in children with TDTs (20). 

Serum ferritin increases linearly with age. WAZ, HAZ, and BMI Z-scores did not correlate with serum ferritin. Stunted children had higher serum ferritin (2, 6, 9-11). However, this study did not show any difference even though the mean serum ferritin was 2742 µg/L compared to Moiz et al. (2017) of 5225 µg/L. Increasing age will increase serum ferritin, but higher serum ferritin will decrease the HAZ (negative correlation) (2,6). The ferritin of 1.107 µg/L cut-off point was responsible for the increased incidence of short stature (9). Hamidah et al., (2008) reported much higher ferritin levels in pediatric TDTs aged 2-13 years who had growth velocity below the 3rd percentile. Serum ferritin weak correlation with growth disorders in pediatric TDTs aged 10-14 years was seen in Bandung, Indonesia. The cut-off ferritin was 3542 μg/L (11). The difference in ferritin cut-off in various studies on the risk of growth retardation in pediatric TDTs may be due to differences in controlling for inclusion and exclusion criteria such as endocrinopathy. Increasing age would decrease the upper/lower segment ratio. The shortening of the trunk segment (vertebral bone) will cause shorth stature. The final height at adulthood in pediatric TDTs was significantly lower (21). The disproportion between upper and lower segments of the body occurred in approximately 15-40% of major thalassemia (22). Spinal growth retardation begins in infancy and worsens progressively on TDTs. Most thalassemia patients, especially men, cannot achieve the targeted height (3). Ferritin correlated the lower body segment and the upper/lower segment ratio. Ferritin levels are closely related to growth disorders in pediatric TDTs, especially short stature and a decrease in the percentile value or HAZ increases (9, 11). Body disproportion in thalassemia is not affected by pubertal or prepubertal only. Pediatric TDTs had decreasing vertebral bone due to inadequate iron chelation and chronic hypoxia. However, this study did not study the role of puberty and endocrinopathy in causing growth retardation. Hypogonadism is one of the determinants of growth failure in pediatric TDTs (1, 3). 

This study did not evaluate other factors contributing to growth retardation in pediatric TDTs. Research on growth retardation in pediatric TDTs should also evaluate endocrinopathy such as hypogonadism and hypothyroidism. At least, the study also pays attention to the status of puberty if the sex hormone examination has problems. Endocrinopathy and chronic hypoxia can cause growth retardation. High ferritin levels indicate poor iron overload control. The weakness of this study also ruled out the side effects of iron chelation and we did not assess the compliance with iron chelation. The number of children who use deferasirox is also less than deferiprone. Growth status on pediatric TDT has a unique pattern in which the decrease in HAZ is more affected than bodyweight that also shortens the upper body segment. Increasing age correlates with increasing serum ferritin followed by decreasing HAZ, especially after ten years old of age. The administration of iron chelating agents also changed growth disorders in pediatric TDT. Serum ferritin had inversed correlation with upper/lower body segment ratio, more severe in serum ferritin is above 2500/L. WAZ and BMI Z-scores were lower in pediatric TDT taking deferiprone than deferasirox. But the opposite was the ratio of upper/lower body segment. Growth retardation was more pronounced in pediatric TDT with high ferritin and in the deferiprone group. Height and upper/lower body segment ratios are more affected.

## References

[1] Skordis N, Kyriakou A (2011). The multifactorial origin of growth failure in thalassaemia. Pediatr Endocrinol Rev.

[2] Moiz B, Habib A, Sawani S (2018). Anthropometric measurements in children having transfusion-dependent beta thalassemia. Hematology.

[3] Kyriakou A, Skordis N (2009). Thalassaemia and aberrations of growth and puberty. Mediterr J Hematol Infect Dis.

[4] Dhouib NG, Ben Khaled M, Ouederni M (2018). Growth and endocrine function in tunisian thalassemia major patients. Mediterr J Hematol Infect Dis.

[5] Tan KA, Lum SH, Yahya A (2019). Prevalence of growth and endocrine disorders in Malaysian children with transfusion-dependent thalassaemia. Singapore Med J.

[6] Pemde H, Jagdish Chandra J (2011). Physical growth in children with transfusion-dependent thalassemia. Pediatric Health Med Ther.

[7] Fahim FM, Saad K, Askar EA, Eldin EN, Thabet AF (2013). Growth parameters and vitamin D status in children with thalassemia major in Upper Egypt. Int J Hematol Oncol Stem Cell Res.

[8] Najafipour F, Aliasgarzadeh A, Aghamohamadzadeh N (2008). A cross-sectional study of metabolic and endocrine complications in beta-thalassemia major. Ann Saudi Med.

[9] Rathaur VK, Imran A, Pathania M (2020). Growth pattern in thalassemic children and their correlation with serum ferritin. J Family Med Prim Care.

[10] Hamidah A, Arini MI, Zarina AL, Zulkifli SZ, Jamal R (2008). Growth velocity in transfusion dependent prepubertal thalassemia patients: results from a thalassemia center in Malaysia. Southeast Asian J Trop Med Public Health.

[11] Fadlyana E, Ma’ani F, Elizabeth M, Reniarti L (2017). Correlation between serum ferritin level and growth disorders in children with thalassemia. Am J Clin Med Res.

[12] Cappellini MD, Cohen A, Porter J, Taher A (2014). Guidelines for the management of transfusion dependent thalassemia (TDT).

[13] Nwosu BU, Lee MM (2008). Evaluation of short and tall stature in children. Am Fam Physician.

[14] Chou JH, Roumiantsev S, Singh R (2020). PediTools electronic growth chart calculators: applications in clinical care, research, and quality improvement. J Med Internet Res.

[15] Arab-Zozani M, Kheyrandish S, Rastgar A, Miri-Moghaddam E (2021). A systematic review and meta-analysis of stature growth complications in β-thalassemia major patients. Ann Glob Health.

[16] Saliba AN, Harb AR, Taher AT (2015). Iron chelation therapy in transfusion-dependent thalassemia patients: current strategies and future directions. J Blood Med.

[17] Azman NF, Abdullah WZ, Mohamad N (2016). Practice of iron chelation therapy for transfusion-dependent thalassemia in Southeast Asia. Asian Biomed.

[18] Panigrahi M, Swain TR, Jena RK, Panigrahi A, Debta N (2020). Effectiveness of deferasirox in pediatric thalassemia patients: Experience from a tertiary care hospital of Odisha. Indian J Pharmacol.

[19] Addis A, Loebstein R, Koren G, Einarson TR (1999). Meta-analytic review of the clinical effectiveness of oral deferiprone (L1). Eur J Clin Pharmacol.

[20] Ballas SK, Zeidan AM, Duong VH, DeVeaux M, Heeney MM (2018). The effect of iron chelation therapy on overall survival in sickle cell disease and β-thalassemia: A systematic review. Am J Hematol.

[21] Soliman AT, Yassin MA, De Sanctis V (2018). Final adult height and endocrine complications in young adults with β-thalassemia major (TM) who received oral iron chelation (OIC) in comparison with those who did not use OIC. Acta Biomed.

[22] Caruso-Nicoletti M, De Sanctis V, Capra M (1998). Short stature and body proportion in thalassaemia. J Pediatr Endocrinol Metab.

